# Supplementary material for: Study on the Localization Technology for Giant Salamanders Using Passive UHF RFID and Incomplete D-Tr Measurement Data
Source: Sensors (Basel). 2025 Dec 23;26(1):106. doi: 10.3390/s26010106 (PMC12787745; doi:10.3390/s26010106)
Supplement: Supplementary file 1 [file sensors-26-00106-s001.zip › File S1. Application Certificate.pdf]

项目应用证明

项目名称： 湖北咸丰忠建河大鲵国家级自然保护区大鲵行为监测研究

项目编号： XF2024070301DN

委托单位： 湖北咸丰忠建河大鲵国家级自然保护区管理中心

承担单位： 湖北民族大学智能科学与工程学院

应用时间： 2024 年 10 月 1 日至 2025 年 7 月 30 日

应用内容说明

由 湖北民族大学 智能科学与工程学院（承担单位）承担的项目《湖北咸丰忠建河大鲵国家级自然保护区大鲵行为监测研究》（项目名称），其研发成果（技术方案）已于 2024 年 10月 1 日起在我单位正式投入应用。

具体应用范围包括：

（1）应用范围： 湖北咸丰忠建河大鲵国家级自然保护区核心区域

（2）解决问题： 解决传统的人工监测方法需要大量的人力与物力资源且存在破坏生态环境的隐患、野生大鲵监测难度高等问题

（3）实现效果： 通过实时传输保护区大鲵监测图频及数据并进行汇总分析，建档保存高清影像，为分析大鲵活动时空分布及生境对大鲵活动影响累计数据基础，提高保护区科研能力。

应用效果评价

该项目的成果在我单位运行稳定，技术指标符合合同要求，有效提升了野生大鲵的监测效果，取得了显著的经济及社会效益。

特此证明！

湖北咸丰忠建河大鲵国家级自然保护区管理中心 42282630016403

委托单位（盖章）：

日期：2025 年 7 月 23 日
