# Supplementary material for: Study on the Localization Technology for Giant Salamanders Using Passive UHF RFID and Incomplete D-Tr Measurement Data
Source: Sensors (Basel). 2025 Dec 23;26(1):106. doi: 10.3390/s26010106 (PMC12787745; doi:10.3390/s26010106)
Supplement: Supplementary file 1 [file sensors-26-00106-s001.zip › File S2. Certificate of No Harm to Giant Salamanders.pdf]

湖北咸丰忠建河大鲵国家级自然保护区

关于【大鲵行为及生境监测研究】相关项目未对野生大鲵造成伤害的证明：

申请人/单位名称：<u>湖北民族大学智能科学与工程学院</u>

身份证号/统一社会信用代码：12422800421956588C

经核实，申请人/单位：湖北民族大学智能科学与工程学院于2024年7月至2026年7月期间，在湖北咸丰忠建河大鲵国家级自然保护区内开展《大鲵行为及生境监测研究》相关项目，研究内容涉及国家二级保护野生动物大鲵（Andrias davidianus）。

根据《中华人民共和国野生动物保护法》及项目实施监管记录，确认如下：

1. 研究已取得合法审批，活动范围、方法符合许可要求；
2. 研究全程接受属地林业部门监管，未对栖息地生态环境造成破坏。

特此证明。

湖北咸丰忠建河大鲵国家级自然保护区管理中心（公章）

2024年7月1日
